# Irinotecan-Induced Gastrointestinal Dysfunction Is Associated with Enteric Neuropathy, but Increased Numbers of Cholinergic Myenteric Neurons

**DOI:** 10.3389/fphys.2017.00391

**Published:** 2017-06-08

**Authors:** Rachel M. McQuade, Vanesa Stojanovska, Elizabeth L. Donald, Ahmed A. Rahman, Dean G. Campelj, Raquel Abalo, Emma Rybalka, Joel C. Bornstein, Kulmira Nurgali

**Affiliations:** ^1^College of Health and Biomedicine, Victoria UniversityMelbourne, VIC, Australia; ^2^Institute of Sport, Exercise and Active Living, Victoria UniversityMelbourne, VIC, Australia; ^3^Australian Institute of Musculoskeletal Science, Western HealthMelbourne, VIC, Australia; ^4^Área de Farmacología y Nutrición y Unidad Asociada al Instituto de Química Médica y al Instituto de Investigación en Ciencias de la Alimentación del Consejo Superior de Investigaciones Científicas, Grupo de Excelencia Investigadora URJC-Banco de Santander-Grupo Multidisciplinar de Investigación y Tratamiento del Dolor, Universidad Rey Juan CarlosAlcorcón, Spain; ^5^Department of Physiology, Melbourne UniversityMelbourne, VIC, Australia

**Keywords:** irinotecan, enteric neuropathy, cholinergic neurons, gastrointestinal dysfunction, chemotherapy

## Abstract

Gastrointestinal dysfunction is a common side-effect of chemotherapy leading to dose reductions and treatment delays. These side-effects may persist up to 10 years post-treatment. A topoisomerase I inhibitor, irinotecan (IRI), commonly used for the treatment of colorectal cancer, is associated with severe acute and delayed-onset diarrhea. The long-term effects of IRI may be due to damage to enteric neurons innervating the gastrointestinal tract and controlling its functions. Balb/c mice received intraperitoneal injections of IRI (30 mg/kg^−1^) 3 times a week for 14 days, sham-treated mice received sterile water (vehicle) injections. *In vivo* analysis of gastrointestinal transit via serial x-ray imaging, facal water content, assessment of gross morphological damage and immunohistochemical analysis of myenteric neurons were performed at 3, 7 and 14 days following the first injection and at 7 days post-treatment. *Ex vivo* colonic motility was analyzed at 14 days following the first injection and 7 days post-treatment. Mucosal damage and inflammation were found following both short and long-term treatment with IRI. IRI-induced neuronal loss and increases in the number and proportion of ChAT-IR neurons and the density of VAChT-IR fibers were associated with changes in colonic motility, gastrointestinal transit and fecal water content. These changes persisted in post-treatment mice. Taken together this work has demonstrated for the first time that IRI-induced inflammation, neuronal loss and altered cholinergic expression is associated with the development of IRI-induced long-term gastrointestinal dysfunction and diarrhea.

## Introduction

A combination of irinotecan (IRI) with 5-fluorouracil (5-FU) and leucovorin (LV) (FOLFIRI) is a common and effective therapy administered to CRC patients (Conti et al., 1996; Saltz et al., 2000). IRI is a semi-synthetic analog of the naturally occurring quinoline alkaloid, camptothecin, and exerts its cytotoxicity via inhibition of topoisomerase I (Top I) triggering S-phase specific cell death (Xu and Villalona-Calero, 2002). Top I is essential for transcription and acts to cut, relax, and reanneal DNA strands. IRI's active metabolite SN-38 binds to Top I and its DNA complex, resulting in the formation of a stable ternary structure that prevents DNA re-ligation and promotes DNA damage and apoptosis (Xu and Villalona-Calero, 2002).

Clinical trials in patients with metastatic CRC have demonstrated a significant survival advantage for FOLFIRI compared with 5-FU/LV alone (Douillard et al., 2000; Saltz et al., 2000), but major dose-limiting toxicities such as neutropenia and chronic diarrhea diminish the clinical efficacy of FOLFIRI treatment (Armand, 1996; Rothenberg et al., 1996; Weekes et al., 2009). Although neutropenia is manageable, IRI-induced diarrhea is typically severe, resulting in hospitalisations, dose-reductions and delays, and termination of treatment in many cases (Swami et al., 2013). Though the prevalence and severity of IRI-induced diarrhea vary greatly depending on regime specifics such as dosage and adjuvant therapies, rates as high as 80% have been reported (Rothenberg et al., 1996).

IRI induces both acute and delayed-onset diarrhea. Acute diarrhea experienced within the first 24 h following IRI administration, occurs in 60–80% of patients (Gibson and Stringer, 2009). Delayed-onset diarrhea arises at least 24 h after IRI administration and occurs in approximately 80% of patients (Saliba et al., 1998). Chemotherapy-induced diarrhea (CID) been linked to early death rates of up to 5% in patients receiving IRI in combination with 5-FU/LV (Rothenberg et al., 2001).

A number of different mechanisms for the gastrointestinal side-effects of IRI treatment have been proposed including luminal accumulation and reactivation of SN-38, sustained disruption to intestinal microflora and continual mucosal damage (Javle et al., 2007; Gibson and Stringer, 2009; Stringer et al., 2009a; McQuade et al., 2014). Although the acute diarrhea is thought to be primarily secretory with attenuation of symptoms by administration of atropine suggesting involvement of cholinergic secretomotor neurons, the underlying mechanism of delayed onset IRI-induced diarrhea is unclear (Gibson et al., 2003; Gibson and Stringer, 2009). We hypothesized that the persistent recurring and long-term gastrointestinal symptoms associated with IRI treatment may result from damage to the intrinsic nervous system of the gastrointestinal tract, the enteric nervous system (ENS). We tested this and found that IRI treatment produced gastrointestinal dysfunction that correlated with loss of enteric neurons, but a substantial increase in the number of cholinergic myenteric neurons, consistent with enteric neuropathy being a major cause of IRI induced diarrhea.

## Methods

### Ethical approval

All procedures were approved by the Victoria University Animal Experimentation Ethics Committee and performed in accordance with the guidelines of the National Health and Medical Research Council (NHMRC) *Australian Code of Practice for the Care and Use of Animals for Scientific Purposes*.

### Animals

Male Balb/c mice aged 6–8 weeks (18–25 g) supplied from the Animal Resources Centre (Perth, Australia) were used for the experiments. Mice had free access to food and water and were kept under a 12 h light/dark cycle in a well-ventilated room at an approximate temperature of 22°C. Mice acclimatized for a minimum of 5 days prior to the commencement of *in vivo* intraperitoneal injections. Mice were euthanized via cervical dislocation. A total of 56 mice were used for this study.

### *In vivo* irinotecan injections

Mice received intraperitoneal injections of IRI (30 mg/kg^−1^) (Sigma-Aldrich, Australia)via a 26 gauge needle, once a day, 3 times a week over a 14 day period to a total of 6 injections. Injections began at Day 0 and continued on Day 2, Day 4, Day 7, and Day 9, the final injection was given on Day 11. IRI was dissolved in sterile water to make 10^−1^ M L^−1^ stock solutions refrigerated at −20°C. The stock was then defrosted and diluted with sterile water to make 10^−2^ M L^−1^ solutions for intraperitoneal injections via a 26 gauge needle. The dose of IRI was calculated to reach a cumulative dose equivalent to a standard human dose in combination therapy, 180 mg/m^2^ per body surface area (Reagan-Shaw et al., 2008; Köhne et al., 2012). Sham-treated mice received sterile water via intraperitoneal injections 3 times a week. The injected volumes were calculated to the body weight; the maximum volume of injected IRI or vehicle did not exceed 200 μL per injection. Mice were euthanized via cervical dislocation at Day 3 (after receiving 2 treatments), Day 7 (after receiving 3 treatments), and Day 14 (after receiving 6 treatments). Post-treatment group mice were euthanized 7 days after the final injection (6 treatments). Colons from all groups were collected for *in vitro* experiments.

A separate cohort of mice was used for food consumption experiments. IRI was dissolved in 0.1% dimethyl sulfoxide (DMSO) (Sigma-Aldrich, Australia) in sterile water to make 10^−2^ M L^−1^ solutions for intraperitoneal injections. Sham-treated mice received 0.1% DMSO in sterile water.

### Gastrointestinal transit

Gastrointestinal transit was studied by X-ray prior to first treatment (day 0) and at 3, 7, and 14 days and 7 days post-treatment of IRI treatment (*n* = 5 mice/group) as described previously (McQuade R. et al., 2016; McQuade R. M. et al., 2016). Briefly, the contrast agent, 0.4 mL of suspended barium sulfate (X-OPAQUE-HD, 2.5 g/mL), was administered via oral gavage. Prior to performing X-ray imaging, animals were trained/conditioned for oral gavage using either 0.9% w/v saline or sterile water (volume 0.1–0.4 ml); this was repeated at least 3 times for each animal with at least 24 h between each training session. Radiographs of the gastrointestinal tract were taken using a HiRay Plus Porta610HF X-ray apparatus (JOC Corp, Kanagawa, Japan; 50 kV, 0.3 mAs, exposure time 60 ms). Mice were immobilized in the prone position by placing them inside a transparent plastic restraint tube with partly open front side for breathing, this comfortably restrains animal movement for a maximum of 1–2 min which is essential for successful X-ray imaging. The training/conditioning with restraint was done by placing the restrainer into the mouse cages at least 24 h prior to the X-ray procedure. X-rays were captured using Fujifilm cassettes (24 × 30 cm) immediately after administration of barium sulfate (T0), every 5 min for the first hour, every 10 min for the second hour, then every 20 min through to 360 min (T360). Animals were closely monitored during and after all procedures. Images were developed via a Fujifilm FCR Capsula XLII and analyzed using eFilm 4.0.2 software. Speed of gastrointestinal transit was calculated as time in min taken to reach each region of the gastrointestinal tract (stomach, small intestines, caecum, and colon). Organ emptying was calculated as the time taken for complete barium emptying from specific gastrointestinal regions (stomach, small intestines) (Cabezos et al., 2008, 2010; Girón et al., 2015).

### Faecal water content and colonic faecal content analysis

Fresh fecal pellets were collected from both sham and IRI-treated mice (*n* = 10 mice/group). Individual mice were placed in holding cages for a period of 15–30 min, the first 5 fresh pellets expelled were collected and weighed immediately to calculate average fresh wet weight. Pellets were then dehydrated for 72 h at room temperature prior to measurement of the dry weight. Water content was calculated as the difference between the wet weight and dry weight. Pellet length was measured in arbitrary units and converted to μm in all X-ray images displaying discernible pellets in the distal colon using an Image J measurement tool. X-ray images were converted to TIFF format and set to 800 × 800 pixels.

### Colonic motility experiments

The entire colon was removed from day 14 and post-treatment sham and IRI-treated mice (*n* = 5 mice/group) and set up in organ-bath chambers to record motor patterns *ex vivo* (Wafai et al., 2013). Briefly, the colon was placed into warmed (35°C), oxygenated physiological saline until the fecal pellets were expelled. The empty colon was cannulated at both ends and arranged horizontally in an organ-bath chamber. The proximal end of the colon was connected to a reservoir containing oxygenated physiological saline to maintain intraluminal pressure. The distal end was attached to an outflow tube that provided a maximum of 2 cm H_2_O back-pressure. Organ baths were continuously superfused with oxygenated physiological saline and preparations were left to equilibrate for 30 min. Contractile activity of each segment was recorded with a Logitech Quickcam Pro camera positioned 7–8 cm above the preparation. Videos (2 × 20 min) of each test condition were captured and saved in *avi* format using VirtualDub software (version 1.9.11).

Recordings were used to construct spatiotemporal maps using in-house edge detection software (Gwynne et al., 2004). Spatiotemporal maps plot the diameter of the colon at all points during the recording allowing contractile motor patterns to be analyzed with Matlab software (version 12). Colonic migrating motor complexes (CMMCs) were defined as propagating contractions directed from the proximal to the distal end of the colon which traveled more than 50% of the colon length (Spencer and Bywater, 2002; Roberts et al., 2007, 2008). Contractions that propagated less than 50% of the colonic length were considered to be short contractions (SCs). Incomplete contractions occurring synchronously at different parts of the colon rather than propagating over the length of the colon were defined as fragmented contractions (FCs) (McQuade R. et al., 2016).

### Histology

The colon was harvested and placed in a 10% formalin solution overnight and then transferred into 70% ethanol the following day. Paraffin embedded colon sections were cut 5 μm thick and deparaffinized, cleared, and rehydrated in graded ethanol concentrations.). To examine the morphological changes to the colon, standard Haematoxylin, and Eosin (H&E) staining protocol was followed (McQuade R. et al., 2016). Ten randomly selected sections per preparation were analyzed and scored on the following parameters: changes in crypt architecture (0–5), reduction in crypt length (0–5), mucosal ulceration (0–5), and immune cell infiltration (0–5) (total score 20) (Rahman et al., 2016). All images were analyzed blindly.

### Immunohistochemistry in cross sections

Colon samples were cut open along the mesenteric border, cleared of their contents, and pinned mucosa up without stretching (*n* = 5 mice/group). Tissues were fixed with Zamboni's fixative overnight at 4°C. Preparations were cleared of fixative by washing 3 × 10 min with DMSO (Sigma-Aldrich, Australia) followed by 3 × 10 min washes with PBS. After washing, tissues were embedded in 100% OCT and frozen using liquid nitrogen (LN_2_) and isopentane (2-methyl butane) and stored in −80°C freezer. Tissues were cut at 20 μm section thickness using a Leica CM1950 cryostat (Leica Biosystems, Germany), adhered to slides and allowed to rest for 30 min at room temperature before processing.

Cross section preparations were incubated with 10% normal donkey serum (Chemicon, USA) for 1 h at room temperature. Tissues were then washed (2 × 5 min) with PBS and incubated with primary antibodies against CD45 (rat, 1:500, BioLegend, Australia), overnight at 4°C. Sections were then washed in PBS (3 × 10 min) before incubation with secondary antibodies labeled with fluorophore donkey anti-rat Alexa 488 (1:200, Jackson Immunoresearch Laboratories, PA, USA) for 2 h at room temperature. The sections were given 3 × 10 min final washes in PBS and then cover slipped using fluorescence mounting medium (DAKO, Australia).

### Immunohistochemistry in wholemount preparations

Segments of the distal colon (2–3 cm) were placed in oxygenated phosphate-buffered saline (PBS) (pH 7.2) containing nicardipine (3 μM) (Sigma-Aldrich, Australia) for 20 min to inhibit smooth muscle contractions (*n* = 5 mice/group). Samples were cut open along the mesenteric border, cleared of their contents, maximally stretched and pinned mucosa down. Tissues were fixed with Zamboni's fixative (2% formaldehyde, 0.2% picric acid) overnight at 4°C. Preparations were cleared of fixative by washing 3 × 10 min with DMSO (Sigma-Aldrich, Australia) followed by 3 × 10 min washes with PBS. Once washed, tissues were dissected mucosa up to expose the myenteric plexus. Fixed tissues were stored at 4°C in PBS for a maximum of 5 days.

Wholemount preparations were incubated with 10% normal donkey serum (Chemicon, USA) for 1 h at room temperature. Tissues were then washed (2 × 5 min) with PBS and incubated with primary antibodies against Protein Gene Product 9.5 (PGP9.5) (chicken, 1:500, Abcam, MA, USA), choline acetyltransferase (ChAT) (goat, 1:500, Abcam, MA, USA) or vesicular acetylcholine transporter (VAChT) (goat, 1:500, Abcam, MA, USA) overnight at 4°C. Tissues were then washed in PBS (3 × 10 min) before incubation with species-specific secondary antibodies labeled with different fluorophores: donkey anti-chicken Alexa 594 (1:200, Jackson Immunoresearch Laboratories, PA, USA) and donkey anti-goat Alexa 488 (1:200, Jackson Immunoresearch Laboratories, PA, USA) for 2 h at room temperature. Wholemount preparations were given 3 × 10 min final washes in PBS and then mounted on glass slides using fluorescent mounting medium (DAKO, Australia).

### Imaging

Wholemount preparations and cross sections were viewed under a Nikon Eclipse Ti laser scanning microscope. Eight randomly chosen three dimensional (z-series) images from each preparation were captured with a x20 objective and processed using NIS Elements software (Nikon, Japan). Fluorophores were visualized using excitation filters for Alexa 594 Red (excitation wavelength 559 nm), Alexa 488 (excitation wavelength 473 nm), and Alexa 405 (excitation wavelength 405 nm). Z-series images were taken at step size of 1.75 μm (1,600 × 1,200 pixels). The number of PGP9.5 and ChAT immunoreactive (IR) neurons was quantified in the myenteric ganglia and CD45-IR cells in cross sections were quantified within a 2 mm^2^ area of each preparation. Quantitative analyses were conducted blindly. The density of VAChT-IR fibers in cross sections of the colon was measured from 8 images per preparation at x20 magnification (total area 2 mm^2^). All images were captured at the same distance from the tissue edges, at identical acquisition exposure-time conditions, calibrated to standardized minimum baseline fluorescence. Minimum baseline fluorescence was determined from the sham-treated tissue, these acquisition settings were used as the acquisition exposure-time for all samples. All images were converted to binary, set to identical thresholds and changes in fluorescence from baseline were measured as mean gray value using Image J software (NIH, MD, USA). All images were analyzed blindly. The density of immunoreactive fibers was then expressed in arbitrary units.

### Statistical analysis

A one-way analysis of variance (ANOVA) with Tukey Kramer *post hoc* test was performed to compare neurochemical and gastrointestinal transit data between multiple groups. Student's unpaired two-tailed *t-*test was used to compare motility, pellet length and fecal water content data. Analyses were performed using Graph Pad Prism (Graph Pad Software Inc., CA, USA). Data are presented as mean ± standard error of the mean (SEM). Value differences were considered statistically significant at *P* < 0.05.

## Results

### Altered gastrointestinal transit following irinotecan administration

**Figure 1:**
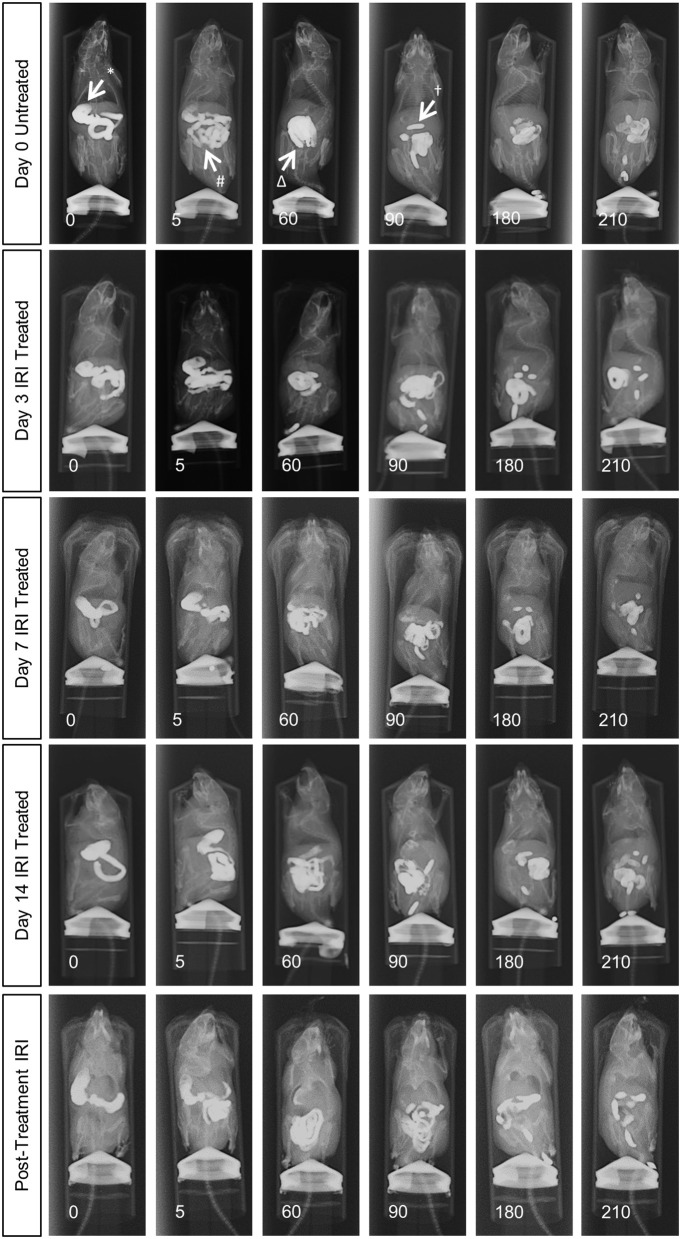
*In vivo* recording of gastrointestinal transit following repeated IRI administration. Representative X-ray images obtained from mice 0 to 210 min after intragastric barium sulfate (0.4mL, 2.5mg/mL) administration. X-ray imaging was performed at day 0 (prior to 1st injection) and following 3, 7, and 14 days of IRI administration and 7 days post-treatment, *n* = 5 mice/group. Stomach (^*^), small intestines (#), caecum (Δ), pellet formation (^†^).

**Figure 2:**
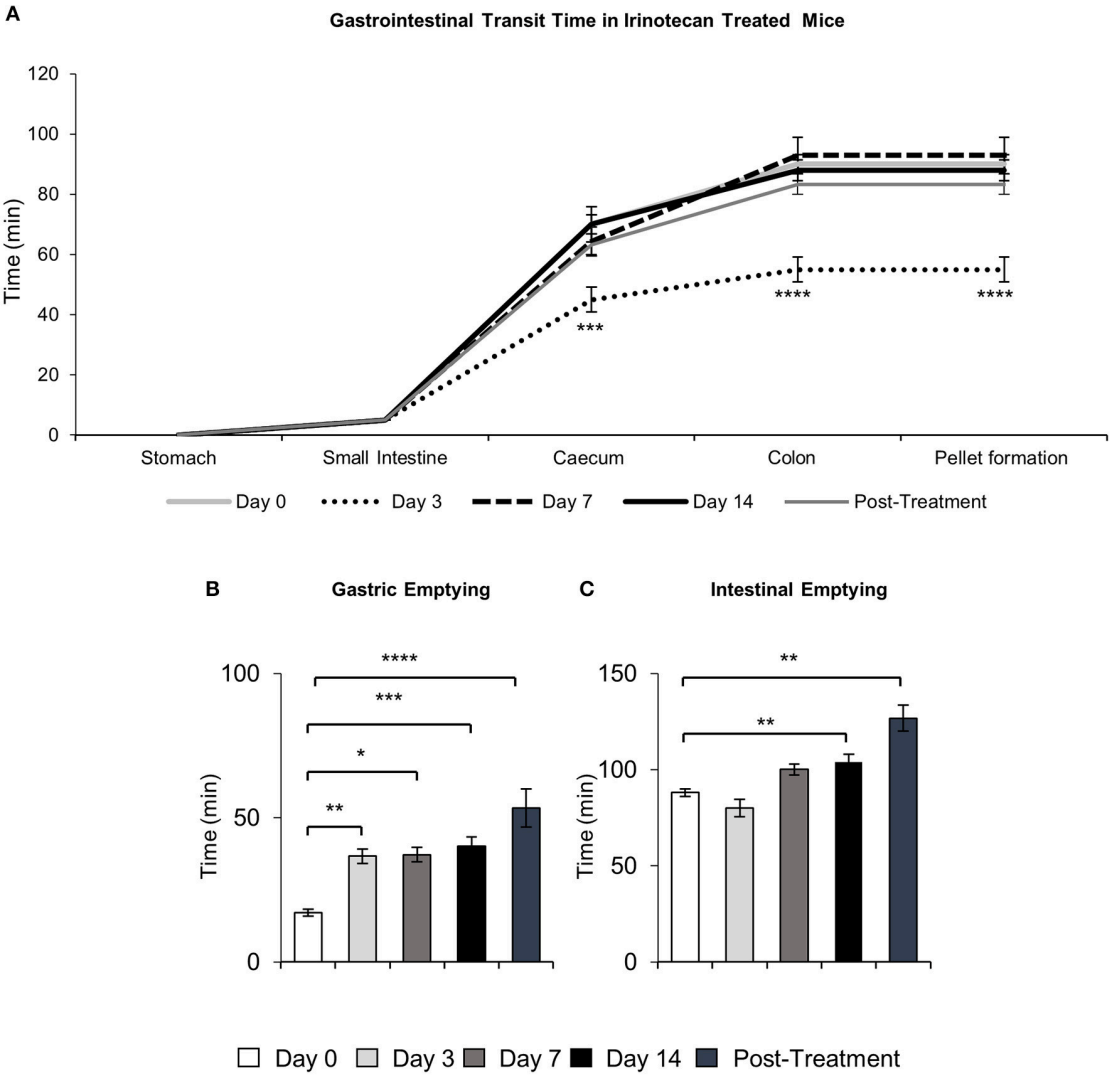
Quantitative analysis of X-ray images. Gastrointestinal transit time, gastric and intestinal emptying following repeated *in vivo* IRI administration. **(A)** Time (min) taken for barium sulfate to reach the stomach, small intestines, caecum and colon before (day 0) and at 3, 7, and 14 days and 7 days post-treatment following IRI administration. **(B)** Time (min) taken for complete emptying of barium from the stomach. **(C)** Time (min) taken for complete emptying of barium from the small intestines. Data presented as mean ± S.E.M. ^*^P < 0.05, ^**^P < 0.01, ^***^P < 0.001, ^****^P < 0.0001 compared to day 0. *n* = 5 mice/group.

To determine the effects of IRI administration on gastrointestinal transit, a series of radiographic images were used to track the movement of barium sulfate through the gastrointestinal tract before the first injection (day 0), after 2 injections (day 3), 3 injections (day 7), 6 injections (day 14 and 7 days post-treatment) (Figure 1). Speed of barium movement was calculated by tracing barium entry from one part of the gastrointestinal tract to the next. Pellet formation time was calculated as the time taken (in minutes) for the first pellet to form. After 3 days of IRI administration, transit was significantly faster to the caecum and colon and pellet formation was significantly quicker (Figure 2A, Table 1). No differences in transit time to the caecum or colon were found at days 7, 14 or post-treatment when compared to day 0 (Figure 2A, Table 1).

**Table 1:** Speed of gastrointestinal transit and emptying following repeated *in vivo* irinotecan administration.

**Parameters measured**		**Day 0**	**Day 3**	**Day 7**	**Day 14**	**Post-treatment**
Speed of transit (time to reach each region, min)	Small Intestines	5 ± 0	5 ± 0	5 ± 0	5 ± 0	5 ± 0
	Caecum	70 ± 3	45 ± 4^***^	64 ± 5	70 ± 6	63 ± 3
	Colon	90 ± 3	55 ± 4^****^	92 ± 6	88 ± 3	83 ± 3
Time for complete barium emptying (min)	Gastric emptying	17 ± 1	36 ± 3^**^	37 ± 2^*^	40 ± 3^***^	53 ± 7^****^
	Intestinal emptying	88 ± 2	80 ± 4	100 ± 7	104 ± 5^**^^††^	126 ± 7^**^^††††^
	Pellet Formation	90 ± 3	55 ± 4^****^	92 ± 6^††††^	88 ± 3^††††^	83 ± 3^††^

* *P < 0.05*,

** *P < 0.01*,

*** *P < 0.001*,

**** *P < 0.0001 significantly different to Day 0*.

†† *P < 0.01*,

†††† *P < 0.0001 significantly different to Day 3, n = 5 per group/time point*.

Although tracing barium movement allowed for the analysis of real time transit speed (the time for barium to reach various parts of the gastrointestinal tract), gastrointestinal organ filling and emptying does not happen simultaneously, therefore we further analyzed the time taken for complete barium emptying from specific gastrointestinal regions (indicative of the gastrointestinal propulsive activity). Significant delays in gastric emptying were found at all time points following IRI treatment and post-treatment (Figure 2B, Table 1). No differences in intestinal emptying were found following 3 or 7 days of IRI administration, but intestinal emptying was significantly delayed following 14 days of IRI treatment, as well as post-treatment, compared to day 0 (Figure 2C, Table 1).

### Colonic fecal content

**Figure 3:**
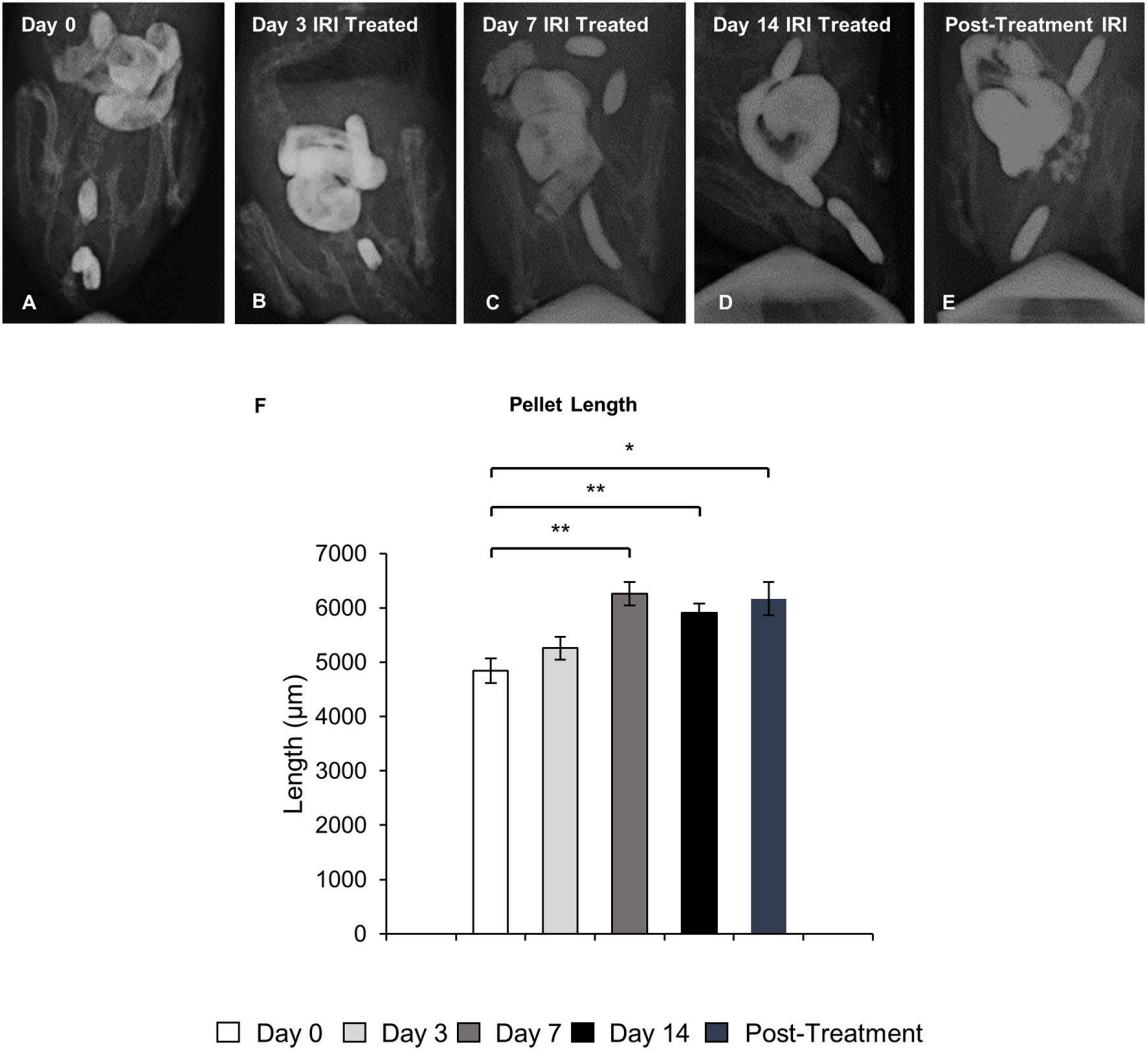
Pellet length following repeated *in vivo* IRI administration. Representative X-ray images of fecal pellets before (day 0) **(A)**, at 3 **(B)**, 7 **(C)**, 14 **(D)** days and 7 days post IRI treatment **(E)**. **(F)** Length (μm) of fecal pellets before (day 0), at 3, 7, 14 days and 7 days post IRI treatment. Data presented as mean ± S.E.M. ^*^P < 0.05, ^**^P < 0.01 compared to day 14 sham, *n* = 5 mice/group.

To define the clinical symptoms resulting from the altered patterns of colonic motor activity, pellet length in X-ray images and fecal water content in freshly collected fecal pellets were analyzed. Pellet length significantly increased after 7 and 14 days of IRI treatment (*P* < 0.01 for both), as well as post-treatment (*P* < 0.05), but no significant differences in pellet length were found following 3 days of IRI treatment when compared to day 0 (Figure 3).

**Figure 4:**
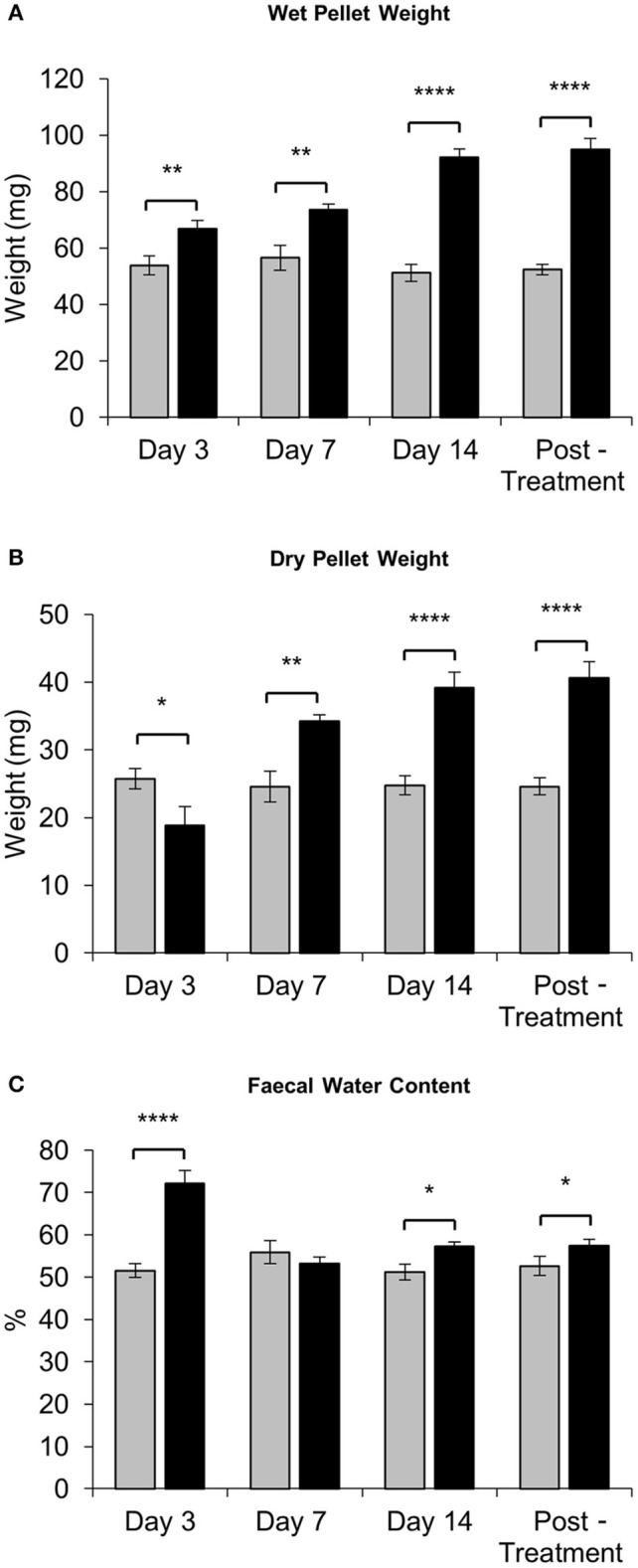
Fecal water content following repeated *in vivo* IRI administration. **(A)** Wet weight of fecal pellets measured immediately upon pellet expulsion. **(B)** Dry weight of fecal pellets measured after 72 h of dehydration at room temperature. **(C)** Faecal water content calculated as the difference between the wet weight and dry weight. ^*^P < 0.05, ^**^P < 0.01, ^****^P < 0.0001, *n* = 10 mice/group.

Fecal water content was calculated as the difference between wet and dry pellet weight. The average wet weight of fresh pellets from IRI-treated mice was significantly greater than that of pellets from sham-treated mice at all time points (day 3 sham: 54 ± 3.4 mg, IRI: 67 ± 2.8 mg, *P* < 0.01: day 7 sham: 57 ± 4.5 mg, IRI: 74 ± 2.0 mg, *P* < 0.01; day 14 sham: 51 ± 2.9 mg, IRI: 74 ± 2.9 mg, *P* < 0.0001; (post-treatment sham: 52 ± 1.9 mg, IRI: 95 ± 3.9 mg, *P* < 0.0001) (Figure 4A). After dehydration, the average dry weight of pellets from IRI-treated mice at day 3 was significantly less than those from sham-treated mice (sham: 26 ± 1.5 mg, IRI: 19 ± 2.7 mg, *P* < 0.05) (*n* = 10 mice/group) (Figure 4B). However, the average dry weight from IRI-treated mice was significantly greater than for pellets from sham-treated mice at day 7 (sham: 25 ± 2.3 mg, IRI: 34 ± 0.9 mg, *P* < 0.01), day 14 (sham: 25 ± 1.4 mg, IRI: 39 ± 2.3 mg, *P* < 0.0001) and post-treatment (sham: 25 ± 1.3 mg, IRI: 41 ± 2.3 mg, *P* < 0.0001) (*n* = 10 mice/group) (Figure 4B). The water content was significantly higher in pellets collected from IRI-treated mice than pellets collected from sham-treated mice at day 3 (sham: 52 ± 1.7%, IRI: 72 ± 3.2%, *P* < 0.0001), day 14 (sham: 51 ± 1.8%, IRI: 57 ± 1.2%, *P* < 0.05) and post-treatment (53 ± 1.3 v, IRI: 57 ± 1.6%, *P* < 0.05) (*n* = 10 mice/group) (Figure 4C). No difference in water content between sham-treated (56 ± 2.7%) and IRI-treated mice (54 ± 1.5%) was found at day 7.

### Changes in colonic motility following irinotecan administration

**Figure 5:**
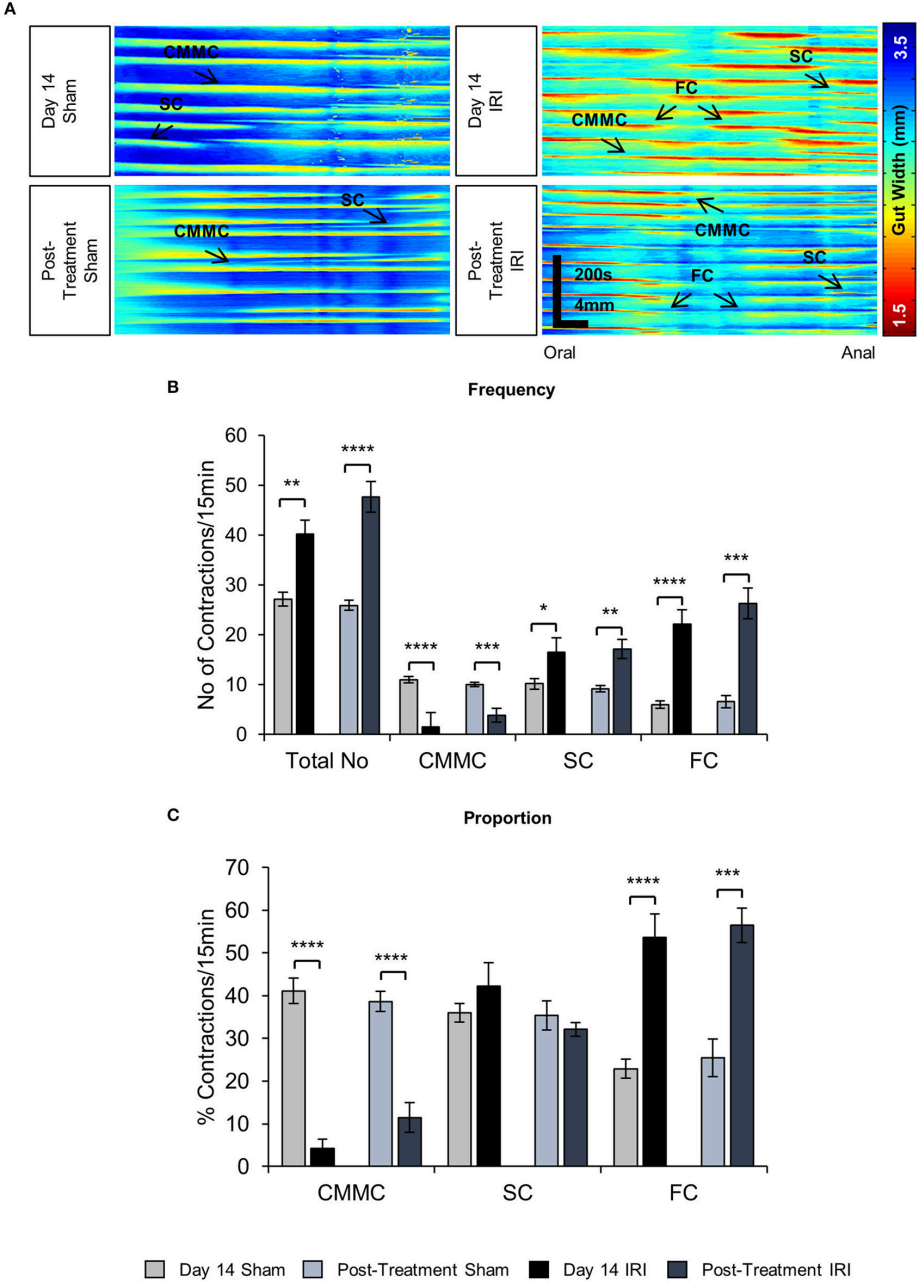
Effects of *in vivo* IRI treatment on the colonic motility. **(A)** Representative spatiotemporal maps generated from digital video recordings of colonic motility from sham and IRI-treated mice. Each contraction can be seen as a reduction in the gut width (red/yellow), while relaxation as an increase in the gut width (blue/green). Colonic migrating motor complexes (CMMCs) propagate >50% of the colon length, short contractions (SCs) propagate <50% of the colon length and fragmented contractions (FCs) are interrupted by period(s) of relaxation during contraction. **(B)** Frequency of contractions including all types of contractile activity and frequency of specific types of contractions in the colons from sham and IRI-treated mice. **(C)** The proportion of CMMCs, SCs and FCs to the total number of contractions. Gray columns: sham-treated, black columns: IRI-treated. Data presented as mean ± S.E.M. *P < 0.05, **P < 0.01, ***P < 0.001, ****P < 0.0001, compared to day 14 sham, *n* = 5 mice/group.

To investigate the effects of IRI on colonic motility controlled by intrinsic innervation, excised colons were studied in organ bath experiments at day 14 of IRI treatment and post-treatment (Figure 5A). The total number of contractions (including all types of motor activity in the colon: CMMCs, short and fragmented contractions) was increased in the colons from day 14 IRI-treated (*P* < 0.01) and post-treatment (*P* < 0.0001) animals compared to sham-treated mice (Figure 5B, Table 2). To determine if this was due to changes in a specific type of motor activity, the frequency and proportion were analyzed for each type of motor contractions.

**Table 2:** Parameters of different types of colonic contractions following repeated *in vivo* irinotecan or sham treatment.

	**Day 14 sham**	**Day 14 IRI**	**Post-treatment Sham**	**Post-treatment IRI**
Total Contractions (per 15 min)	27.7 ± 1.8	41.1 ± 1.9^**^	25.9 ± 1.0	47.7 ± 3.1^****^
Frequency CMMCs (per 15 min)	11.0 ± 0.9	1.6 ± 0.7^****^	10.0 ± 0.4	3.8 ± 1.4^***^
Proportion CMMCs (%)	40.6 ± 2.5	4.6 ± 2.1^****^	38.7 ± 2.4	11.5 ± 3.5^****^
Frequency SCs (per 15 min)	10.2 ± 1.8	15.6 ± 1.8^*^	9.1 ± 0.7	17.2 ± 1.9^**^
Proportion SCs (%)	35.8 ± 3.6	38.2 ± 3.9	35.4 ± 3.5	32.1 ± 1.6
Frequency FCs (per 15 min)	6.0 ± 1.16	23.8 ± 2.5^****^	6.6 ± 1.2	26.7 ± 3.1^***^
Proportion FCs (%)	23.7 ± 2.3	57.3 ± 4.4^****^	25.4 ± 4.4	56.4 ± 4^***^

* *P < 0.05*,

** *P < 0.01*,

*** *P < 0.001*,

**** *P < 0.0001, significantly different to Day 14 sham-treated mice, n = 5 per group/time point*.

A decrease in the frequency and proportion of CMMCs was observed in the colons from day 14 IRI-treated compared to sham-treated animals (*P* < 0.0001 for both) (Figures 5B,C, Table 2). The decrease in frequency (*P* < 0.001) and proportion (*P* < 0.0001) of CMMCs persisted in the colons from post-treatment mice compared to sham-treated animals (Figures 5B,C, Table 2).

Following 14 days treatment with IRI, the frequency of SCs in the colon was greater compared to sham-treated colon (*P* < 0.05) (Figure 5B, Table 2), but no significant difference in the proportion of SCs was found in day 14 IRI-treated compared to sham-treated mice (Figure 5C). Similarly, the frequency of SCs in the colon increased in post-treatment mice compared to sham-treated mice (*P* < 0.01) (Figure 5B, Table 2), but without a change in the proportion of SCs in post-treatment mice when compared to sham-treated mice (Figure 5C).

Both the frequency and the proportion of FCs was significantly higher in IRI-treated mice when compared to sham-treated mice (*P* < 0.0001 for both) (Figures 5B,C, Table 2). Similarly, the frequency (*P* < 0.001) and proportion of FCs (*P* < 0.001) was significantly greater in the colon from post-treatment IRI mice than in sham-treated mice (Figures 5B,C, Table 2).

### Mucosal damage and intestinal inflammation following irinotecan administration

**Figure 6:**
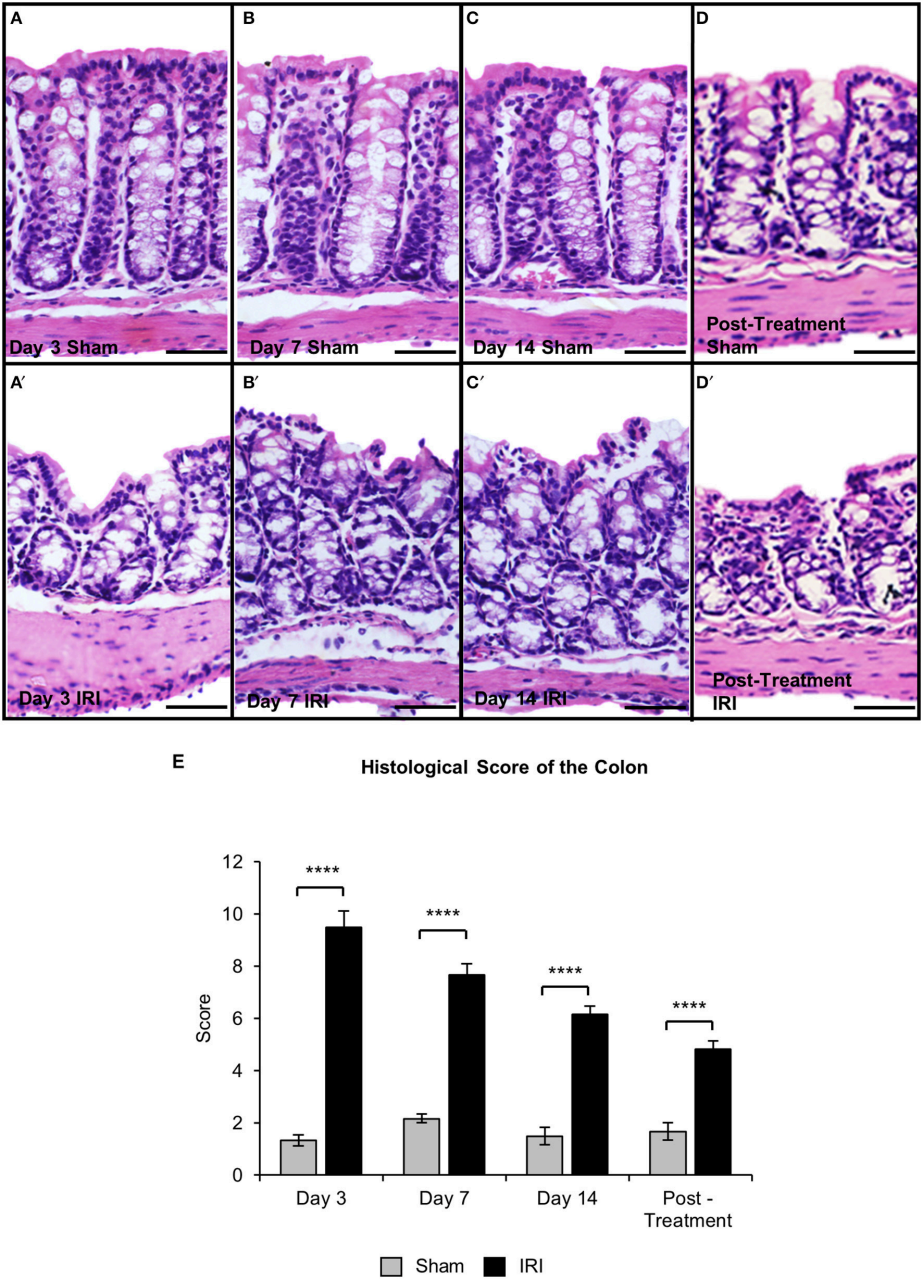
Gross morphological change in the colon following repeated *in vivo* IRI administration. H&E staining in the colon from sham-treated and IRI-treated mice at 3 **(A,A′)**, 7 **(B,B′)**, 14 **(C,C′)** days, and 7 days post-treatment **(D,D′)**. Scale bar = 100 μm. Histological scoring of morphological changes in the colon **(E)**. Data presented as mean ± S.E.M. ^****^P < 0.0001, *n* = 5 mice/group.

Histological structure of the colons from the sham-treated animals at days 3, 7, 14, and post-treatment appeared healthy with a visible brush border as well as uniform crypts (total histological score = 5) (Figures 6A–D,E). Severe mucosal ulceration and crypt distension was observed in the colon from the IRI-treated group at day 3 (total histological score = 17) (Figures 6A′,E). Colon sections from IRI-treated mice at days 7 (total histological score = 12) (Figures 6B′,E) and 14 (total histological score = 12) (Figures 6C′,E) displayed severe mucosal ulceration coupled with crypt hypoplasia and disorganization, which were not observed in colonic preparations from sham-treated mice. Colon sections from post-treatment IRI mice displayed crypt hypoplasia and disorganization, but the epithelial brush border was visible and appeared to be restored (total histological score = 8) (Figures 6D′,E).

**Figure 7:**
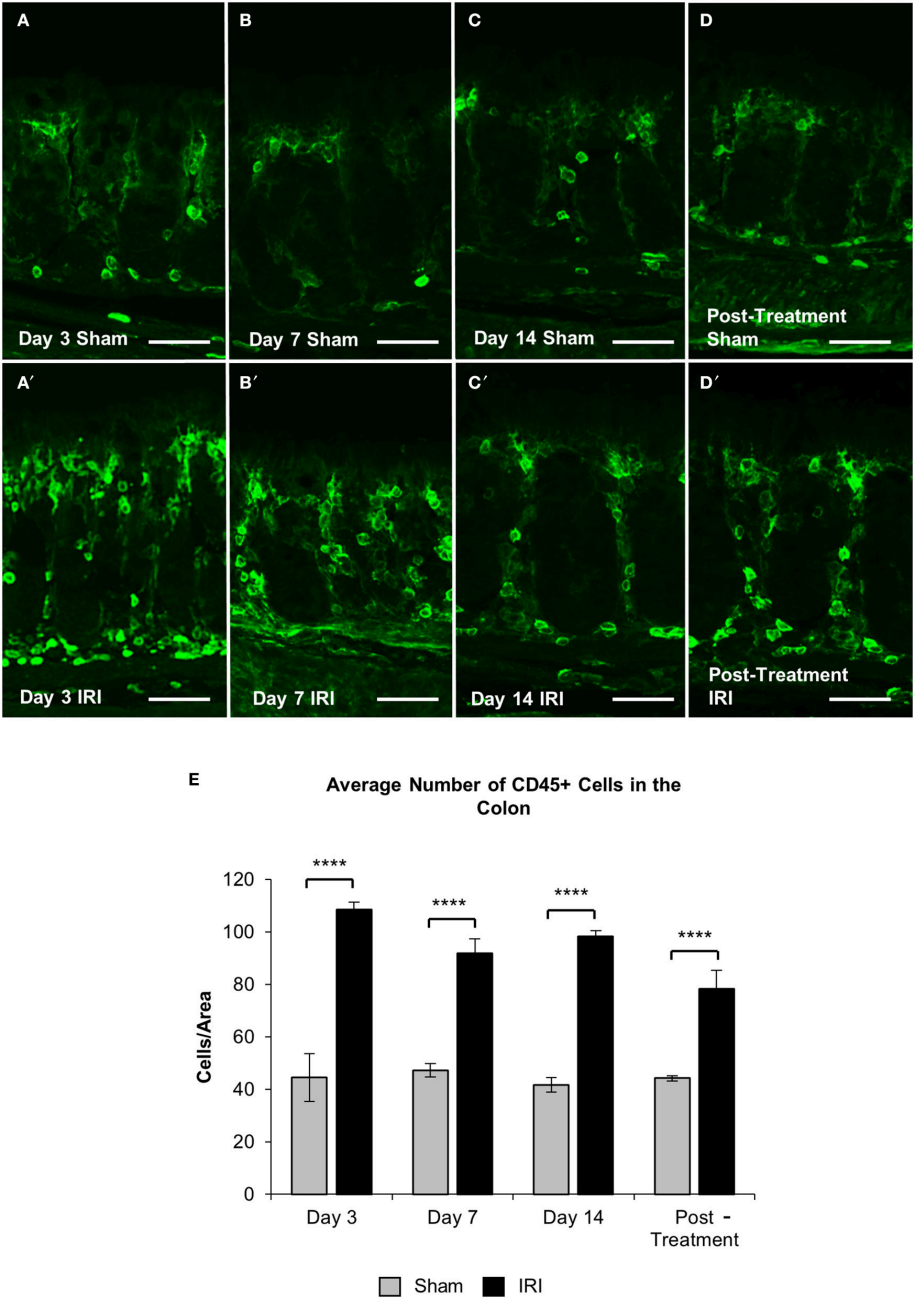
CD45+ leukocytes in the colon. Cross sections of the colon labeled with a leukocytes marker anti-CD45+ antibody (green) from sham-treated and IRI-treated mice at 3 **(A,A′)**, 7 **(B,B′)**, 14 **(C,C′)** days, and 7 days post-treatment **(D,D′)**. Scale bar = 100 μm. Number of CD45+ cells was counted within 2 mm^2^ of the mucosa in the colon sections from sham and IRI-treated mice at 3, 7, and 14 days, and 7 days post-treatment **(E)**. Gray columns: sham-treated, black columns: IRI-treated mice. Data represented as mean ± S.E.M. ^****^P < 0.0001, *n* = 5 mice/group.

To investigate if acute and chronic IRI treatment causes inflammation, immune cell infiltration in the colon was analyzed. Immune cells in colonic cross sections were labeled with a pan-leukocyte marker anti-CD45 antibody following 3, 7, 14 days and post IRI treatment compared to sham-treated mice (Figures 7A–D′). Total numbers of CD45 positive cells were counted within a 2 mm^2^ area. A significant increase in the number of CD45 positive cells was found in the colon following 3 (sham: 45 ± 9; IRI: 109 ± 3, *P* < 0.0001), 7 (sham: 47 ± 3; IRI: 92 ± 5, *P* < 0.0001), and 14 days (sham: 42 ± 3; IRI: 98 ± 2, *P* < 0.0001) of IRI administration when compared to sham treatment. This increase persisted in post-treatment IRI mice (92 ± 7, *P* < 0.0001) compared to sham (44 ± 1) group (Figure 7E).

### Neuronal loss and changes in cholinergic neurons and fibers following repeated *in vivo* administration of irinotecan

**Figure 8:**
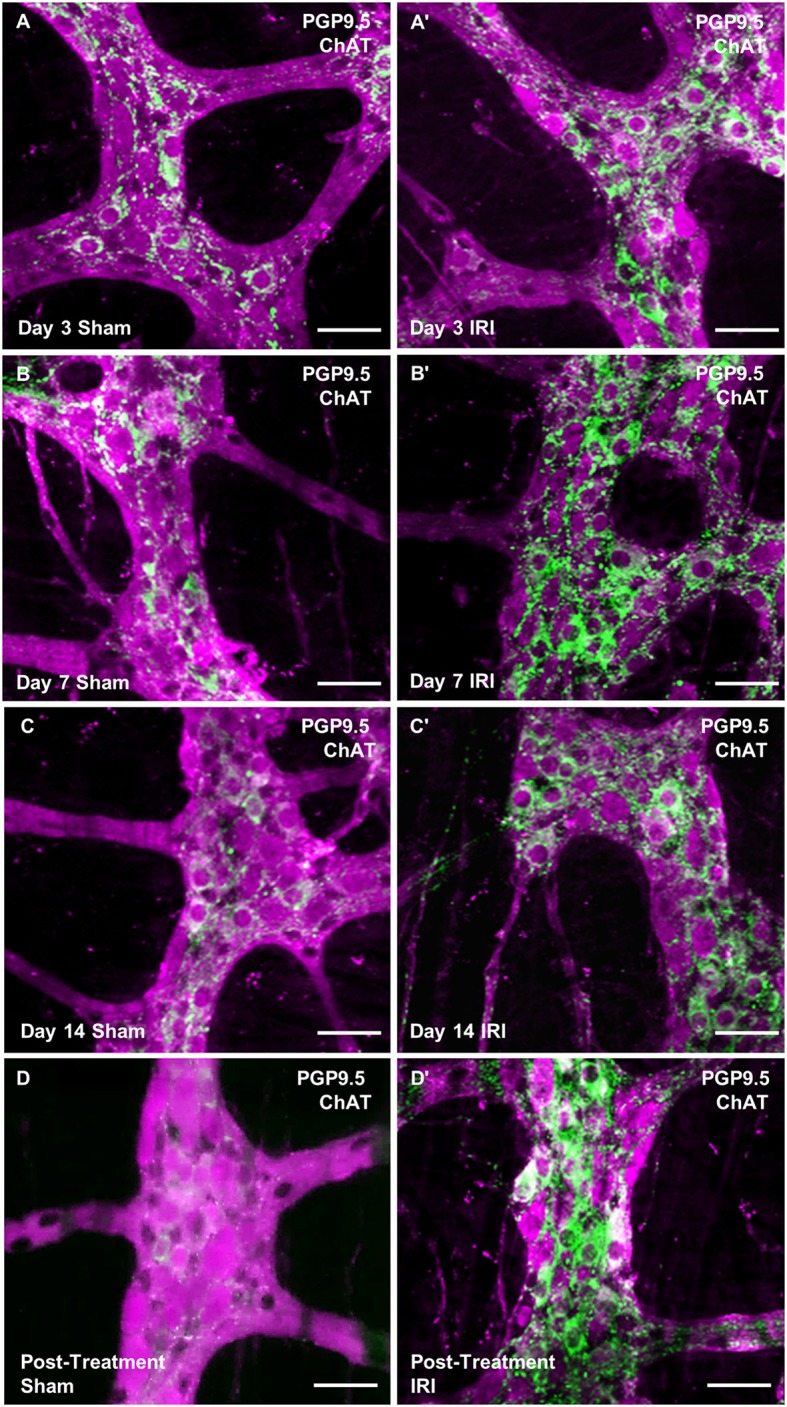
Wholemount preparations of myenteric plexus. Myenteric neurons were labeled using a pan-neuronal marker anti-PGP9.5 antibody (magenta) and cholinergic neurons were labeled using anti-ChAT antibody (green) in the wholemount preparations of the colon from sham-treated and IRI-treated mice at 3 **(A,A′)**, 7 **(B,B′)**, 14 **(C,C′)** days and 7 days post-treatment **(D,D′)**. Scale bar = 50 μm.

**Figure 9:**
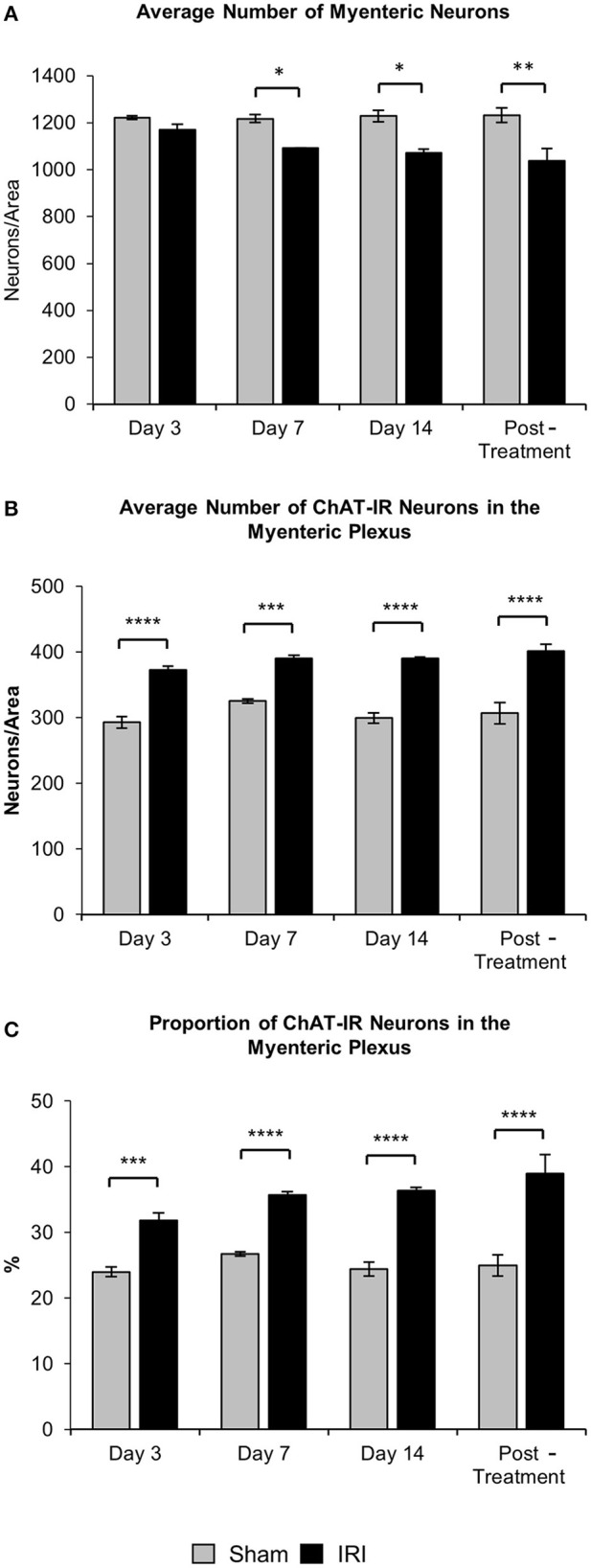
Effect of repeated *in vivo* IRI administration on the total number of neurons and average number and proportion of ChAT-IR myenteric neurons. **(A)** Average number of PGP9.5-IR neurons in the colon was counted per 2 mm^2^ at 3, 7, and 14 days, and 7 days post-treatment in both sham and IRI-treated mice. **(B)** Average number of ChAT-IR neurons in the colon was counted per 2 mm^2^ at 3, 7, and 14 days and 7 days post-treatment in both sham and IRI-treated mice. **(C)** Proportion of ChAT-IR neurons calculated to the total number of PGP9.5-IR myenteric neurons in the colon per 2 mm^2^ at 3, 7, and 14 days and 7 days post-treatment in both sham and IRI-treated mice. Gray columns: sham-treated, black columns: IRI-treated mice. Data presented as mean ± S.E.M. ^*^P < 0.05, ^**^P < 0.01, ^***^P < 0.001, ^****^P < 0.0001, *n* = 5 mice/group.

To investigate changes to the total number of myenteric neurons wholemount preparations were labeled with a pan-neuronal marker anti-PGP9.5 antibody (Figure 8). Repeated *in vivo* administration of IRI induced myenteric neuronal loss when compared to the sham-treated groups at days 7 (sham: 1,218 ± 17; IRI: 1,092 ± 2, *P* < 0.05) and 14 (sham: 1,262 ± 34; IRI: 1,072 ± 23, *P* < 0.05) (Figure 9A). This neuronal loss persisted in mice post IRI treatment (sham: 1,232 ± 32; IRI: 1,038 ± 52, *P* < 0.01). No significant difference in numbers of myenteric neurons was found at day 3 (sham: 1,222 ± 9; IRI: 1,171 ± 22) (Figure 9A).

To determine if IRI administration was associated with changes in a specific subpopulation of myenteric neurons, the average number and proportion of neurons immunoreactive (IR) for ChAT specific to cholinergic neurons was analyzed in both sham and IRI-treated mice (Figure 8). Repeated *in vivo* administration of IRI induced a significant increase in the average number of ChAT-IR neurons when compared to sham-treated group at days 3 (sham: 293 ± 9; IRI: 372 ± 6, *P* < 0.0001), 7 (sham: 325 ± 3; IRI: 390 ± 5, *P* < 0.001), 14 (sham: 320 ± 15; IRI: 390 ± 3, *P* < 0.0001) and post-treatment (sham: 306 ± 15; IRI: 401 ± 10, *P* < 0.0001) (Figure 9B). The proportion of ChAT-IR neurons significantly increased at all time points (Figure 9C).

**Figure 10:**
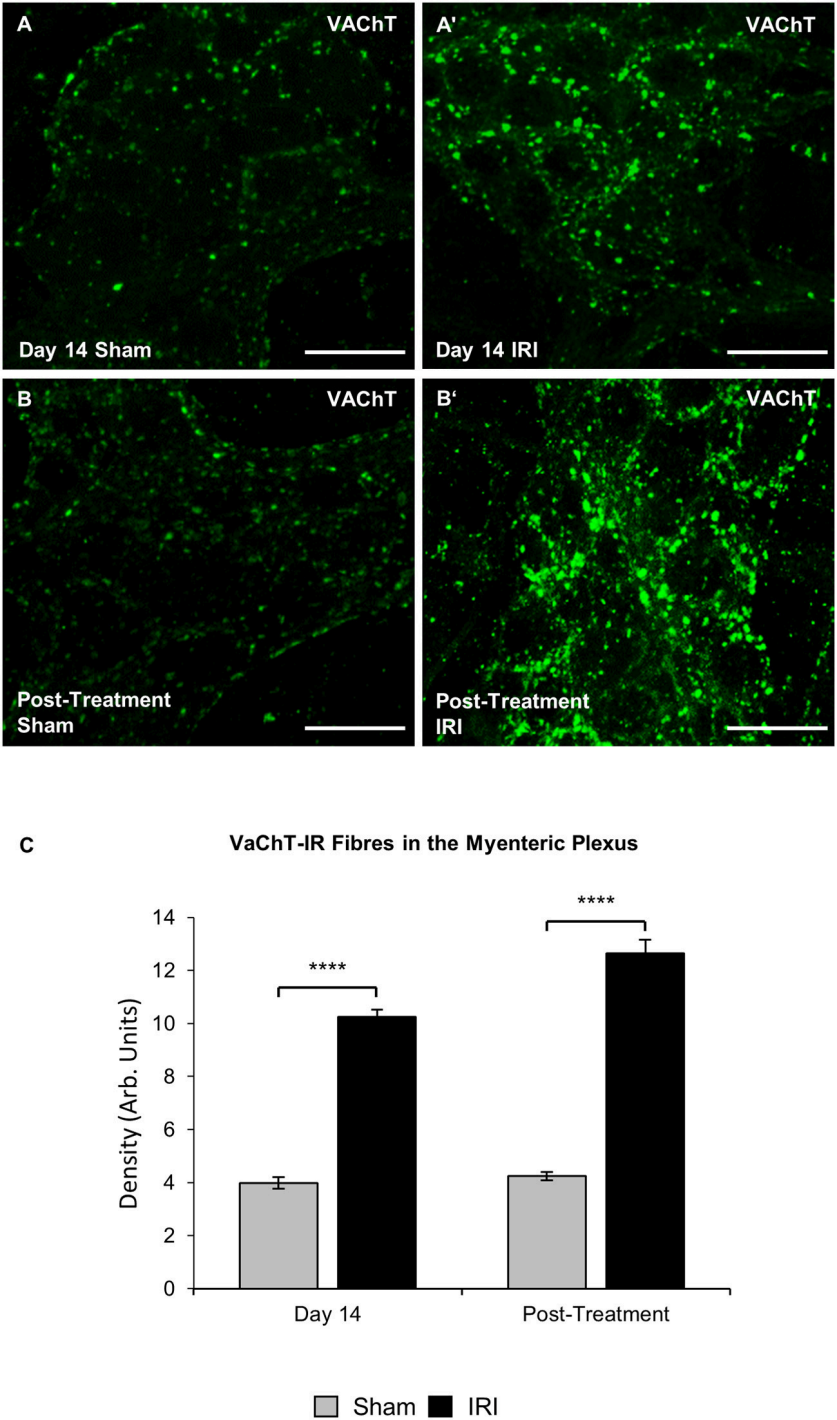
Effect of repeated *in vivo* IRI administration on the density of VAChT-IR cholinergic fibers in the myenteric plexus. Wholemount preparations of VAChT-IR (green) fibers in the myenteric ganglia of the colon from sham and IRI-treated mice at 14 days **(A,A′)** and 7 days post-treatment **(B,B′)** Scale bar = 25 μm. **(C)** Density of VAChT-IR fibers in the myenteric plexus was analyzed at 14 days and post-treatment in both sham and IRI-treated mice. Gray columns: sham-treated, black columns: IRI-treated mice. Data presented as mean ± S.E.M. ^**^P < 0.01, ^****^P < 0.0001, *n* = 5 mice/group/time point.

The define whether increase in ChAT expression in neuronal cell bodies was due to the increased synthesis or decreased transport and release of acetylcholine, cholinergic fibers were labeled within myenteric ganglia in wholemount preparations of the colon using anti-VAChT antibody at day 14 and post IRI treatment (Figures 10A–B'). Repeated *in vivo* administration of IRI induced a significant increase in the density of VAChT-IR fibers in the myenteric plexus at day 14 (sham: 4.0 ± 0.2; IRI: 10.3 ± 0.3, *P* < 0.0001) and post-treatment compared to the sham-treated group (sham: 4.2 ± 0.2; IRI: 12.6 ± 0.5, *P* < 0.0001) (Figure 10C).

## Discussion

This study is the first to investigate IRI-induced enteric neuropathy, colonic motility, and gastrointestinal function in mice. The results show that changes in intestinal transit and increased motor activity are associated with symptoms of diarrhea evidenced by increased pellet length and fecal water content. Although repeated administration of IRI causes severe mucosal damage to the murine colon throughout the experimental period, post-treatment colons showed signs of epithelial regeneration. However, increased leukocyte infiltration was found both during the treatment period and after 7 days post-treatment. Increased intestinal transit time taken together with very high fecal water content at day 3 of IRI administration indicate that at this time point diarrhea had significant secretory component, whereas unchanged intestinal transit time, delayed intestinal emptying and long pellets with increased water content observed at later time points as well as significant changes in colonic motility at day 14 were associated with delayed onset/chronic diarrhea. This study is the first to demonstrate a distinct difference in both the pathophysiology and functional consequences of acute and delayed onset/chronic IRI-induced diarrhea. Acute diarrhea associates with significant increases in the number of ChAT-immunoreactive neurons and the density of cholinergic nerve fibers observed at Day 3 of IRI administration leading to increased secretion, whereas symptoms of chronic diarrhea at days 7, 14, and post-treatment may result from the damage and loss of enteric neurons observed at these time points leading to impaired motility. Taken together our results implicate intestinal inflammation, neuronal loss and phenotypic changes with increased numbers of cholinergic neurons and fibers in the pathogenesis of irinotecan-induced gastrointestinal dysfunction underlying diarrhea.

Gastrointestinal mucositis is a frequent and debilitating complication resulting from the systemic effects of cytotoxic chemotherapy and the local effects of radiotherapy (Avritscher et al., 2004). Inflammation, epithelial degradation, and intestinal ulceration, manifesting as mucositis, are well-established consequences of IRI administration (Duncan and Grant, 2003; Sonis et al., 2004; Stringer et al., 2009a,b). Although the incidence and severity of mucositis varies greatly according to patient characteristics and treatment regimens, the prevalence of IRI-induced gastrointestinal mucotoxicity in the form of secretory diarrhea has been reported to be as high as 87% (Rougier et al., 1998; Avritscher et al., 2004). Our results show severe mucosal ulceration, crypt hypoplasia and disorganization in the colon following both short and long-term IRI treatment. A significant increase in CD45+ leukocytes in colonic mucosa observed following 3 days of IRI administration persisted throughout the course of treatment as well as post-treatment in our study. This is in line with previous reports for the rat jejunum and colon following IRI treatment, where villus blunting, epithelial atrophy and crypt ablation were reported (Logan et al., 2008). However, in our study, mucosal regeneration is evident in post-treatment mice in which colonic crypts are still disorganized, but the epithelial brush border has recovered. Acute intestinal toxicity associated with anti-cancer treatment is believed to result from crypt cell death, which triggers mucosal inflammation and breakdown of the intestinal mucosal barrier, however controversy exists regarding whether this is a direct result of cytotoxicity or is mediated through a series of intermediate events (Sonis et al., 2004).

The basic pathophysiology of mucositis may be broken into 5 sequential phases (i) initiation; (ii) up-regulation and message generation; (iii) signaling and amplification; (iv) ulceration and inflammation; and (v) healing (Sonis et al., 2004; Lee et al., 2014). In the healing phase, proliferation and differentiation of the gastrointestinal epithelium returns approximately 2 weeks post-chemotherapy (Sonis et al., 2004; Lee et al., 2014). Mucosal regeneration has been confirmed in human studies showing that early histological changes in the gastrointestinal tract following chemotherapeutic administration are resolved within days of treatment cessation, with no abnormal endoscopic findings in patients as early as 16 days post-chemotherapeutic treatment (Keefe et al., 2000). However, although histological damage has resolved, long-term diarrhea following chemotherapy treatment persists up to 10 years post-treatment (Schneider et al., 2007; Denlinger and Barsevick, 2009; Numico et al., 2015). Similar results have been found in pelvic radiation therapy, during which intestinal permeability and mucosal injury peak mid-treatment then gradually improve, while nausea, diarrhea and abdominal pain persist throughout the course of treatment (Carratù et al., 1998; Hovdenak et al., 2000). It has been suggested that crypt cell death in radiation syndrome is triggered indirectly by apoptotic endothelial lesions (Paris et al., 2001), highlighting that intestinal dysfunction at least in part, may be a consequence of an indirect cascade of events mediated by non-epithelial tissues (Sonis et al., 2004).

Although the underlying mechanisms of delayed onset and long-term diarrhea remain unclear, early mucosal damage, and acute intestinal inflammation can lead to death and damage of enteric neurons resulting in long-term gastrointestinal dysfunction (Boyer et al., 2005; Linden et al., 2005; Nurgali et al., 2007, 2011). Our study is the first demonstrating that repeated administration of IRI results in myenteric neuronal loss of up to 16% from day 7 to post-treatment. Given its cell cycle specificity, IRI is believed to have relatively no toxic effect on differentiated post-mitotic cells such as neurons. However, the IRI analog camptothecin administered at concentrations that inhibit Top-I has been found to induce apoptosis in cortical neurons (Morris and Geller, 1996). Despite the significant loss of myenteric neurons at Days 7 and 14 which persisted 7 days post-treatment, both the absolute number and the proportion of ChAT-IR neurons in the myenteric plexus were increased as early as 3 days following IRI treatment, with increases of up to 17% persisting throughout all experimental time-points and in post-treatment mice. The increased proportion of ChAT-IR neurons might be due to either a preferential loss of other neuronal sub-types with preferential sparing of ChAT-IR neurons or to phenotypic changes in myenteric neurons. However, the increase in the absolute number of ChAT-IR neurons alongside neuronal loss can only be due to phenotypic changes in myenteric neurons leading to increased ChAT expression as opposed to preferential loss of other neurons. Furthermore, it has been shown previously that IRI binds to the active site of acetylcholinesterase resulting in functional inhibition of the enzyme and increased persistence of extracellular acetylcholine (Dodds and Rivory, 1999; Harel et al., 2005).

To determine if the increase in ChAT-IR neurons was accompanied by increased cholinergic innervation of the muscle, we quantified the density of cholinergic fibers containing VAChT, a transporter responsible for transferring acetylcholine into vesicles. IRI-treated mice had significantly higher densities of VAChT-IR fibers at day 14 and post-treatment. The increase in VAChT-IR is consistent with the possibility that IRI induces increased numbers of cholinergic motor neurons; however, the sprouting or new branches of pre-existing cholinergic motor neurons as well as increased density of extrinsic VAChT-IR fibers cannot be excluded. Moreover, whether increased synthesis of acetylcholine occurs only in cholinergic neurons making them easier to identify or whether non-cholinergic neurons start expressing ChAT needs to be further determined. In addition, the increase in the proportion of ChAT neurons may, in part, be due to a selective loss of other neuronal subtypes. Further analysis of other neuronal subtypes including nNOS-IR inhibitory motor neurons is warranted.

Taken together, these data suggest that IRI induces increase in enteric neuronal acetylcholine synthesis and release as well as reduced degradation leading to an excessive amount of acetylcholine, known as cholinergic syndrome, affecting physiological functions of the gut. Cholinergic neurons are a vital component of the excitatory motor innervation of the colon (Furness, 2012). Acetylcholine is a major excitatory neurotransmitter responsible for the contractions of circular and smooth muscle (Furness, 2012) and mucosal secretion (Cooke, 2000). Acute diarrhea experienced within the first 24 h of IRI treatment has been attributed to cholinergic syndrome in colorectal cancer patients (Hecht, 1998). Delayed onset and long-term diarrhea are, however, believed to be multifaceted (Bleiberg and Cvitkovic, 1996; Saliba et al., 1998). Our results demonstrated increased intestinal transit accompanied by faster pellet formation at day 3 of IRI treatment. Although intestinal transit returned to sham levels at later time points, the increased motor activity in the colon associated with symptoms of diarrhea (increased pellet length and fecal water content) persisted at both day 14 and post-treatment. It has previously been shown that colonic propulsion speed correlates positively with pellet length (Costa et al., 2015). Our data indicate increased colonic propulsive activity and presence of diarrhea in IRI-treated mice. The total number of contractions following IRI administration was significantly higher due to increases in both the number and proportion of short and fragmented contractions. Short and segmenting contractions play a central role in the formation of productive motor patterns in the healthy intestine (Gwynne et al., 2004). Short distance contractions result in segmentation of the colon, which is essential for mixing and absorption of colonic contents (Huizinga and Chen, 2014). Chemotherapy-induced neuronal loss in the myenteric plexus has been previously shown in mice and rats following administration of various agents. Downstream effects on colonic motility and gastrointestinal transit following administration with platinum compounds cisplatin and oxaliplatin (Vera et al., 2011; Wafai et al., 2013; Pini et al., 2016) have been associated with increased proportions of nNOS neurons and oxidative stress in enteric neurons, whilst administration with fluoropyrimidine 5-fluorouracil has been correlated with intestinal inflammation (McQuade R. et al., 2016; McQuade R. M. et al., 2016). These findings highlight that enteric neuropathy may be a critical component in the development of long-term chemotherapy-induced gastrointestinal dysfunction.

In conclusion, this study demonstrates that IRI treatment induces mucosal damage and inflammation which may contribute to neuronal loss and phenotypic changes in the myenteric plexus. Increased expression of ChAT-IR neurons and VAChT-IR nerve fibers in the myenteric plexus may underlie alterations in colonic motor activity and gastrointestinal transit following IRI treatment resulting in chronic gastrointestinal dysfunction. This study is the first to show that neuropathic changes in the gut may be key players in the manifestation of delayed-onset and long-term chronic diarrhea that persists after the cessation of IRI treatment.

## Author contributions

RM, conception and design, data acquisition, analysis and interpretation of data, manuscript writing; VS, data acquisition, analysis and interpretation, manuscript revision; ED, interpretation of data, manuscript revision; AR, interpretation of data, manuscript revision; DC, data acquisition, analysis and interpretation, RA, interpretation of data, manuscript revision; ER, interpretation of data, manuscript revision; JB, interpretation of data, manuscript revision; KN: conception and design, interpretation of data, supervision of the study, manuscript revision. KN and AR obtained funding. All authors approved final version of the manuscript.

### Conflict of interest statement

The authors declare that the research was conducted in the absence of any commercial or financial relationships that could be construed as a potential conflict of interest.

## Abbreviations

5-FU: 5-fluorouracil
ChAT: choline acetyltransferase
CID: chemotherapy-induced diarrhea
CRC: colorectal cancer
CMMC: colonic migrating motor complex
ENS: enteric nervous system
FC: fragmented contraction
FOLFIRI, 5-fluorouracil: leucovorin and irinotecan
IR: immunoreactive
IRI: irinotecan
LV: leucovorin
PGP 9.5: Protein Gene Product 9.5
SC: short contraction
Top I: topoisomerase I
VAChT: vesicular acetylcholine transporter.
